# Acoustic projectors make covert bioacoustic chirplet signals discoverable

**DOI:** 10.1038/s41598-023-29413-2

**Published:** 2023-02-14

**Authors:** Paolo Casari, Jeff Neasham, Guy Gubnitsky, Davide Eccher, Roee Diamant

**Affiliations:** 1 CNIT and Department of Information Engineering and Computer Science, University of Trento, Trento, 32123 Italy; 2 School of Engineering, Newcastle University, Newcastle upon Tyne, NE1 7RU UK; 3 Department of Marine Technology, University of Haifa, Haifa, 3498838 Israel

**Keywords:** Electrical and electronic engineering, Biomimetics

## Abstract

To disguise man-made communications as natural signals, underwater transceivers have the option to pre-record animal vocalizations, and play them back in a way that carries meaningful information for a trained receiver. This operation, known as biomimicking, has been used to perform covert communications and to emit broadband signals for localization, either by playing pre-recorded animal sounds back into the environment, or by designing artificial waveforms whose spectrum is close to that of bioacoustic sounds.However, organic sound-emitting body structures in animals have very different trans-characteristics with respect to electro-acoustic transducers used in underwater acoustic transceivers. In this paper, we observe the distortion induced by transmitting pre-recorded animal vocalization through a transducer’s front-end, and argue that such distortion can be detected via appropriate entropy metrics. We test ten different metrics for this purpose, both via emulated transmission and in two field experiments. Our result indicate which signals and entropy metrics lead to the highest probability of detecting transducer-originated distortions, thus exposing ongoing covert communications. Our research emphasizes the limitations that man-made equipment incurs when reproducing bioacoustic sounds, and prompts for the choice of biomimicking signals that are possibly suboptimal for communications or localization, but help avoid exposing disguised transmissions.

## Introduction

The advance in underwater acoustic communications for security applications has promoted the development of low-probability-of-detection (LPD) techniques^1,2^, where the goal is to avoid that an interceptor detects the transmitted signal. Common LPD approaches use frequency spreading to hide the signal below the noise floor (e.g., see Baek et al.^3^). However, considering the narrow frequency band of underwater acoustic communications and the availability of low-power high performance computing, finding the spreading sequence is feasible, even by means of an exhaustive search. To circumvent this issue, recent approaches emerged that employ biomimicking communications as an alternative means to achieve LPD.

In biomimicking communications, the signal is disguised as the vocalization of marine mammals, e.g., by encoding information into sounds that imitate dolphin and seal whistles and clicks, or whale songs. This solution enables high-power transmissions, while an interceptor device can be misled into believing that received sounds are not a modulated information carrier, but rather mammal vocalizations. The survey of Qiao et al.^4^ makes a systematic review of biomimicking approaches available until 2018, including performance figures such as achieved bit rates and ranges. The authors conclude that biomimicking is a promising technique, but further research is needed towards more efficient modulation schemes, camouflage improvement, as well as encryption functionalities, possibly based on real animal vocalization recorded in the wild. In the following, we provide a brief literature overview exploring how to disguise communications via marine fauna vocalizations and transient sounds.

As a first contribution dating back to 2008, Dol et al.^5^ present the results of a sea trial where information was transferred via modulated cetacean sounds, albeit resulting in non-negligible error rates. Liu et al.^6^ suggest to transmit dolphin whistles for synchronization and to encode information in the time separation between dolphin clicks. ElMoslimany et al.^7^ generate non-linear frequency-modulated signals based on the time-frequency content of dolphin whistles, and encode information in their amplitude, frequency modulation rate and duration. The authors achieve uncoded bit error ratios (BER) lower than 1% using real acoustic channels measured during the KAM’11 experiment campaign. Along the same line, Liu et al.^8^ create artificial copies of real dolphin whistles, and propose to send the original whistle as a synchronization signal, while further copies carry information. Using a time-reversal mirror, the system covers a range of 5.5 km with a BER lower than 10^−3^ in a controlled sea trial.

Ahn et al.^9^ mimic dolphin whistles using a continuous-frequency antipodal modulation scheme. Tests in simulations and sea experiments show good BER with error correction coding, and a good level of mimicry. Jiajia et al.^10^ encode the information to be transmitted into the inter-click intervals of killer whale vocalizations used to localize targets. Similarly, Ahn et al.^11^ propose to encode bit sequences in the transition between different types of mimicked dolphin whistles, and resort to machine learning to identify different whistles in the spectrogram of a received acoustic signal. Bilal et al.^12^ consider a set of four humpback whale songs and use them to build a Morse code-like sequence of sounds to represent different English alphabet letters. One additional acoustic preamble enables synchronization and channel estimation based on the matching pursuit algorithm. A recent contribution by Xie et al.^13^ proposes to smooth the time-frequency contour of bottlenose dolphin vocalizations, and to subdivide them into segments. Information is then modulated into each segment via a frequency shift in the corresponding spectrogram. In the same vein, it has been proposed to encode information in continuous-phase signal models of realistic cetacean whistles^14^, although recent results suggest that similar schemes may actually expose a biomimicking signal to a trained receiver^15^. In the same way biomimicking communications help covertness, the playback of sperm whale songs can help active sonars remain undetected. While Jiang et al. propose to employ sperm whale call pulses as a sonar signal^16^, Sun et al.^17^ re-engineer a sperm whale click sequence through time hopping and frequency hopping, in order to disguise sonar-like sequences with better detection properties amidst actual (played back) click sequences. The approach of Liu et al.^18^ combines biomimicking communications and low-probability of detection principles by superimposing a whale sound to an information-carrying direct-sequence spread spectrum (DSSS) signal. The receiver leverages the whale signal for channel estimation and recovers the DSSS signal through virtual time-reversal techniques. Finally, Qiao et al.^19^ design a modem that automatically selects dolphin sounds from a local database, and encodes information in the time interval between subsequent transmitted sounds.

Considering the above communication system designs, we observe that current interception techniques mostly focus on detecting weak signals, and not on telling biomimicking communications apart from real bio-acoustic signals. Conversely, in this work we propose a scheme to classify detected mammal vocalization-like signals into one of two classes: real signals and biomimicking signals. We base our scheme on the fact that these two types of signals are emitted from very different emitters: the former through the vocalization system of a marine mammal, the latter through a mechanical system, typically an electro-acoustic transducer.

Modern transmitting circuitry can synthesize a very realistic replica of a recorded signal via a high-speed, high-precision digital-to-analog converter and a linear amplifier. For instance, the EvoLogics modem working in the 18–34 kHz band^20^ provides a sampling rate of 250,000 samples per second and a resolution of 16 bits per sample, and the 7–17 kHz modem relies on a sampling rate of 62,500 samples per second. Both modems yield a very low quantization noise thanks to a much higher-than-Nyquist sampling frequency. However, converting the replica into the equivalent acoustic signal in the water is more challenging. Typical piezo-ceramic transducers are inherently narrowband devices and, even with substantial damping or the use of piezo-composite technology, they cannot match the very broadband response of a marine mammal’s vocalization system. Consequently, an anthropogenic signal will generally lose some of its random statistical properties compared to a real mammal’s vocalization. Such loss of randomness can be quantified using entropy metrics. In other words, we argue that real mammal vocalizations are different from biomimicking signals, especially because of much higher peak-to-average-ratio and phase irregularities. This diversity can be measured by computing the entropy of the signal. Our work informs biomimicking system design based on engineered as well as played-back animal sounds by evaluating how the emitted signals really resemble natural sounds. In fact, time-based signal modulations^10,17,19^, frequency-based modulations^9,13^, and even direct playback^12,16^ can be put in jeopardy if the last element of the transmitter chain (i.e., the transducer) exposes the emitted signal as a non-natural one.

In the next sections, we first present our system model and entropy metrics, then we introduce the model of an electro-acoustic transducer front-end and the parameters that drive its response. We proceed by describing the results of our emulations and field experiments (one in a lake and one in the Mediterranean sea), before discussing conclusions from our study.

## Materials and methods

To present our approach, we first introduce our assumptions and entropy metric definitions; then, we proceed with a model for underwater acoustic projectors, and with the description of the biomimicking signals we consider.

### Assumptions

Our working assumption in this paper is that a device is trying to pass communications covertly using signals similar to real underwater fauna vocalizations. To achieve this, the transmitter has previously recorded real animal sounds, and has prepared them for transmission through appropriate analog front-ends (e.g., via resampling, frequency up- or down-shifting, and out-of-band filtering). We also assume that the covert transmitter is sufficiently advanced to avoid unnatural sound patterns that would not be compatible with typical animal communications. Therefore, in order to detect the ongoing covert communication, we need to leverage different signal characteristics. In particular, we argue that analog transducer front-ends distort the vocalizations, and that we can detect the change in the signals by comparing the original transmitted signals with recordings taken close to the transmitter.

While biomimiking is possible for both chirplet signals (whistles) and click signals, we argue that the latter are much harder to mimic. This is because of the broad bandwidth of the click signal, which makes is hardly reproducible by any acoustic transducer. The resulting low-pass effect makes the emitted signal easy to detect as non-biological. Therefore, relatively narrowband whistle signals are a much better candidate for biomimicking communications. We thus focus our work on these signals.

### Entropy metrics

We now introduce the concept of entropy and explain how we choose a suitable entropy metric to discriminate between biomimicking and natural vocalizations. The key assumption in our work is that we can detect the inherent differences between an acoustic projector and the vocalization system of a marine animal specimen through entropy metrics. In particular, we explore the Renyi entropy, the sample entropy, the transfer entropy and its normalized version, the mutual entropy, the differential entropy, the approximate entropy, two instances of the vector entropy, and the Tsallis entropy. For the definition and an effective summary of the characteristics of these metrics, we refer the reader to Namdari and Li^21^.

We choose these metrics in our work since they capture and quantify the fluctuations within a given signal. In particular, the Renyi entropy^22^ generalizes the Shannon entropy through a user-defined parameter, α,

1 $$\begin{aligned} S_\alpha =\frac{1}{1-\alpha } \; \ln \left( \sum \limits _{k=1}{n}p_k^\alpha \right) , \end{aligned}$$

where $p_k^\alpha$ is the probability distribution of the *k*th random variable. Thus, the Renyi entropy takes the form of the Shannon entropy for discrete variables for $\alpha =1$, of the collision entropy (for testing if two datasets intersect) for $\alpha =2$, and of the min-entropy (which measures the information within a time series) for $\alpha \rightarrow +\infty$.

The sample entropy^23^ measures the fluctuations in the data between sequential time windows, and is defined by the conditional probability that two windows of observations of the same size are equal up to a tolerance *r*. Thus, sample entropy can be used to detect rapid changes within the signal, which are likely in a real bioacoustic signal, but less likely in a biomimicking signal. The transfer entropy^24^, detects the information transfer between different systems to detect if the variability in one system can explain the variability in the other:

2 $$\begin{aligned} T_{Y\rightarrow X}(m,l)=\sum p(x_1,\ldots x_m,y_{m-l+1},\ldots ,y_m)\cdot \log _2\frac{p(x_{m+1}|x_1,\ldots ,x_m,y_{m-l+1},\ldots ,y_m)}{p(x_{m+1}|x_1,\ldots ,x_m)}, \end{aligned}$$

where $x_i$ and $y_i$ are discrete variables of processes *X* and *Y*, and *m* and *l* are the number of past observations in *X* and *Y*, respectively.

Mutual entropy^25^ measures the uncertainty reduction in a time series compared to another time series:

3 $$\begin{aligned} I(X;Y)=-\sum \limits _{x\in X,y\in Y}p(x,y)\cdot \log _2\frac{p(x,y)}{p(x)p(y)}. \end{aligned}$$

Differential entropy^26^ is a causality test that determines whether a certain time series helps predict the future dynamics of another time series. Approximate entropy^27^ measures patterns in time series, by quantifying the logarithmic likelihood that sequences of patterns that are close for *m* observations remain close on next comparisons as well. As such, it uncovers their regularities by extracting the noise from the original data. The measure does not depend on an estimate of the signal’s probability density function, and can detect when the signal’s regularity breaks. The vector entropy^28^ quantifies the inequalities between the entropy values for different data subsets. Finally, the Tsallis entropy^29^ tests if the correlations within these subsets are local or general:

4 $$\begin{aligned} S_q=\dfrac{\displaystyle 1-\sum \limits _{i=1}{n}p_i^\alpha }{q-1}. \end{aligned}$$

### Acoustic projector model

**Figure 1:**
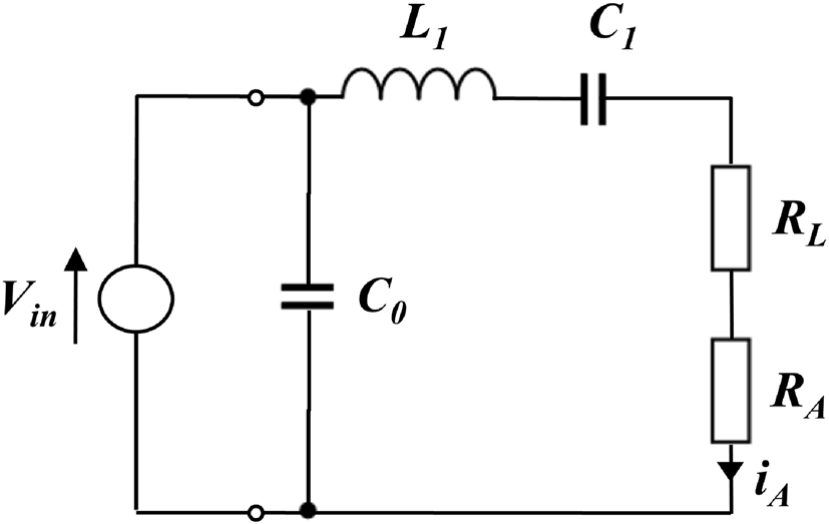
Equivalent circuit of an acoustic projector based on a piezoelectric electro-acoustic transducer.

In this subsection, we present an electrical equivalent circuit to model the conversion from a voltage waveform (drive) to an acoustic (i.e., pressure) waveform in the water. We will exploit this model to investigate the effects of a typical acoustic projector on a transmitted biomimicking signal. The circuit in Fig. 1 models a piezoelectric transducer (PZT) with a single mechanical resonance. It is acknowledged that this may constitute an oversimplified model of the tubular or spherical transducer elements often used in acoustic modems, which typically have more than one mode of resonance in play when a broadband signal is being transmitted. However, while more complex equivalent circuit models can be constructed, we observed that even this simplistic model yields encouraging agreement with experimental data.

**Figure 2:**
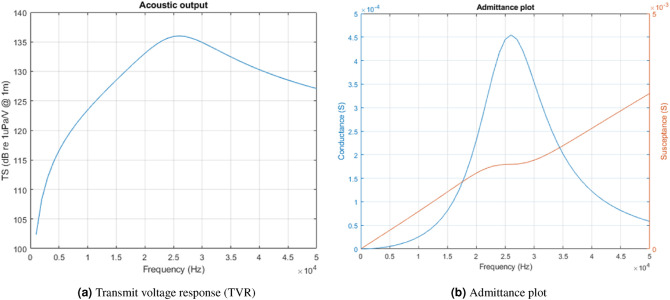
Typical transmit voltage response and admittance plot for the acoustic projector model presented in Fig. 1.

The resonant frequency of the modelled transducer is controlled by the values of $L_1$ and $C_1$, which form a series resonant circuit. $V_{in}$ is the applied drive voltage, represented here by an ideal voltage source. $C_0$ represents the static capacitance of the transducer element, which is commonly tuned out in practical drive circuits by either series or parallel inductance, to reduce the reactive power that must be supplied. The bandwidth of the modelled transducer is controlled by varying the values of the total series resistance of $R_L+R_A$, which control the degree of damping of the resonant circuit, and hence the *Q*-factor. The ratio of $R_A$ to $R_L$ determines the transmitting efficiency of the transducer, where $R_L$ determines the dissipated power (loss), and $R_A$ the radiated power (sound). We remark that, in any event, the efficiency of the transducer does not influence the signal parameters of interest in the paper. Figures 2a and 2b show an example response of a modelled transducer, with parameters chosen to give a good approximation of the acoustic modem transducers used for the experiments. ($C_0$ = 11 nF, $C_1$ = 1500 pF, $L_1$ = 25 mH, $R_L$ = $R_A = 1.1$ k$\Omega$, resulting in a resonant frequency of 26 kHz and bandwidth of 8 kHz.) This device is typical of transducers used in modern commercial modem products and it is practically difficult to achieve lower *Q*-factor with current technology. However, the model is also used to simulate transducers with widely varying resonance and bandwidth, as shown Table 1, to explore the limits of the proposed entropy technique.

We simulate the reception of biomimicking communication signals by integrating the characteristics of the modeled transducer with existing recordings of marine mammal vocalizations. Specifically, we filter a vocalization signal segment using a rational transfer function^30^ defined by the numerator and denominator coefficients of the transducer’s acoustic transfer function derived from the above circuit model.

We consider different settings for the parameters of the transducer model, resulting in seven different transfer functions with different resonant frequency, bandwidth, and shape. To represent a practical PZT that is typically designed to operate at 50% efficiency, we set $R_L=R_A$. In addition, to provide a realistic predominance of a reactive load over most of the frequency range, the choice of the parameter series $L_1$ and $C_1$ ensures that the peak susceptance is always higher than the peak conductance. All the model’s parameter combinations determined for the simulations and their corresponding transfer function characteristics (i.e., bandwidth and resonant frequency) are listed in Table 1.

**Table 1:** Realistic transducer model parameters considered in this work, and their corresponding transfer function parameters used to simulate the transmission of biomimicking signals.

$C_0$ (nF)	$C_1$ (pF)	$L_1$ (mH)	$R_L=R_A$ (k$\Omega$)	Bandwidth (kHz)	Resonant frequency (kHz)
11	750	37	2.35	20.3	30.2
			3.2	27.5	
		50	3.48	22.2	26
		80	4.65	18.5	20.5
	1500	25	0.55	7	26
			1.1	14	
			2.2	28	

### Vocalizations used as a test case

We now describe the sounds emitted by different common marine mammals, which we consider as a test case to quantify how the limitations of the acoustic projectors influence a covert communicator’s capability to disguise its own structured communications as marine fauna sounds. Specifically, we select a dolphin whistle, and vocalizations by a beaked whale, an orca, a humpback whale, and a sea lion. This selection of signals covers a diverse set of spectral characteristics, including different concentrations of the acoustic power over the signal band. For example, the dolphin whistle is broadband, and has a clear spectral pattern including a down- and up-sweeping section of different duration. The beaked whale sound is mostly multi-tonal, except for a short up-down sweep about 150 ms after the start of the signal. The orca also emits a multi-tonal sound, with fewer spectral components than the beaked whale, and characterized by a short initial up-sweep. The spectrum of the sea lion vocalization is comparatively richer; however, the signal is time-paced, and includes silence intervals lasting about 120 ms. Finally, the humpback whale vocalization has a broad spectral footprint, with several closely-spaced tones covering the whole bandwidth of the transducer, and does not exhibit any significant sweeps.

## Results

In this section, we discuss the outcomes of our emulation study and of our field experiments. The main purpose of our analysis is to exploit entropy measures to tell apart original marine fauna vocalizations from their biomimicking version emitted by the transducers. For this purpose, we define the *entropy ratio* for two entropy measures $H_1$ and $H_2$ as

5 $$\begin{aligned} \rho = \frac{|H_1-H_2|}{|H_1|+|H_2|}. \end{aligned}$$

This normalized ratio quantifies how close the two given entropy measures are.

**Figure 3:**
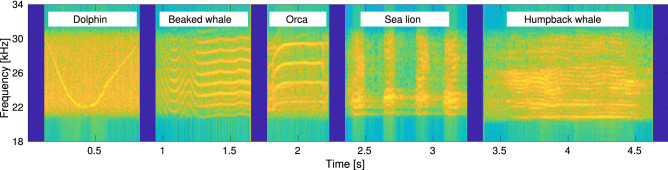
Spectrograms of five bioacoustic signals considered in the emulation analysis and in the lake experiment.

In the following, we discuss the results of three experiments where we analyze the differences between a recording and a playback of marine mammal vocalization. The first experiment is an emulation, where we apply a transducer model and simulate its response to above considered signals. The second experiment is a lake trial, where we played back the signals in a lake environment. The third is a sea experiment. To explore the performance of different types of modems, for the emulation we considered seven different transducers’ models, whose resonant frequency is between 20 kHz and 30 kHz. Similarly, in the lake and sea experiments, we used different modems, one working in the frequency band of 18 kHz to 34 kHz and one working in the 2 kHz to 20 kHz bands, respectively. Therefore, for the emulation and lake experiment setups, we shifted the signals in the frequency domain to match the operational band of the transducers. Conversely, in the sea experiment, we played back a set of original signals. The time-frequency representation of these two versions is given in Figs. 3 and 8 later on, respectively.

### Emulated biomimicking signal transmissions

**Figure 4:**
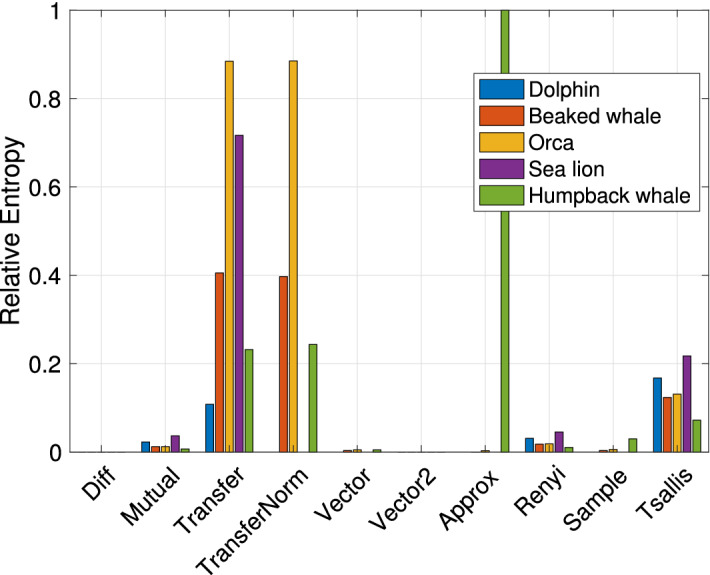
Emulation. Relative entropy values obtained using different entropy metrics, for each of the five considered bioacoustic signals. The results are averaged over the seven projector parameter sets in Table 1.

We start by analyzing our emulation results involving actual biomimicking signals transmitted through a modeled acoustic transducer. In Fig. 4, we show entropy ratio values using each of the entropy metrics introduced in the previous section, for all five examined signals. Each histogram bar conveys the average taken across all of the nine parameter sets of the projector reported in Table 1, and different bars refer to different animal sounds. Bars are grouped by the entropy metric employed to compute the relative entropy as per Eq. (5).

We observe that the best separation is obtained by using the transfer entropy. To compute this metric, we first divide the time series of both the original and the transducer-emitted signal samples into blocks $B_1, B_2, \ldots , B_{N_b}$ of length 0.04 s and feed the entropy calculation with the current block, $B_i$, the previous block, $B_{i-1}$, and the block before it, $B_{i-2}$. Then, we average the entropy ratio over all blocks. The Transfer Entropy measures how related blocks $B_i$ and $B_{i-1}$ are, by quantifying how predicting $B_i$ to be a combination of $B_{i-1}$ and $B_{i-2}$ is better than predicting $B_i$ based only on $B_{i-1}$. Because of this operation, the transfer entropy measures how correlated (or, in other words, how smooth) the time series is. The fact that such a metric leads to a large entropy ratio, when applied to the original animal sound and its biomimicking version transmitted by the transducer, supports our claim that the transducer-emitted time series is more predictable than the original one. For example, from Fig. 4 we observe that the entropy ratio (5) for most considered vocalizations is above 0.2 when using the transfer entropy. This high value means that the two entropies of the real and mimicked signals can be well distinguished.

**Figure 5:**
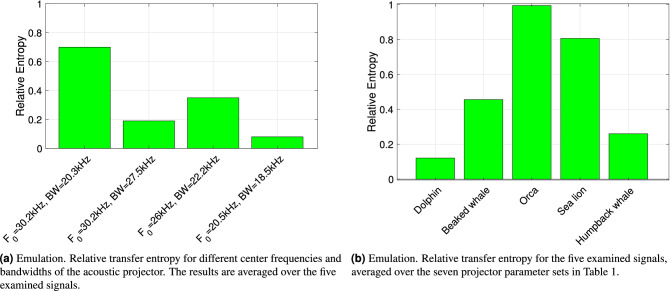
Discrimination performance of the relative transfer entropy as a function (**a**) of the transducer parameters and (**b**) of the type of vocalization. Different transducer models and vocalizations lead to different degrees of separation between a real marine mammal vocalization and a biomimicking signal, but telling the two signals apart is possible in all cases.

We now examine the sensitivity of the interceptor as a function of the system’s parameters, i.e., the signal’s bandwidth as determined by the resistance values $R_L$ and $R_A$, and the signal’s frequency band as determined by the inductance $L_1$. Figure 5a shows the relative entropy of the original and transducer-emitted signal, computed using the Transfer Entropy metric, and averaged over the five examined signals, as listed in Table 1. The best capability to separate the original signal from the biomimicking one are obtained for parameter set index 1, which relates to a carrier frequency of 30.2 kHz and a bandwidth of 20.3 kHz. This is also the smallest of the three bandwidth values tested here: as a lower transducer bandwidth leads to a heavier smoothing of transmitted signals, these results further support our claim that a biomimicking signal becomes easier to detect using entropy metrics when the transducer’s transfer function makes the emitted sound more predictable.

It is also interesting to investigate which animal vocalization is more suited to biomimicking among those considered in this paper, and which leads to emitted signals that are easier to detect as imitations of real marine fauna sounds. For this, we compute the relative entropy between each signal and each transducer-emitted version of that signal. In doing so, we average out the influence of the projector by computing the mean relative entropy value over the nine realistic transducer parameter sets of Table 1. The results in Fig. 5b show that among the signals tested in our work the best biomimicking signal is the dolphin whistle, whereas the worst is the orca vocalization. Observing the structure of these signals as shown by the spectrogram in Fig. 3, we observe that the orca sound we transmitted tends to settle on the same portion of the spectrum for a longer time, whereas the chosen dolphin whistle is a broadband, frequency-varying signal. This leads to greater waveform variability, which partly compensates the transducer’s smoothing effect, and thus reduces the relative entropy.

**Figure 6:**
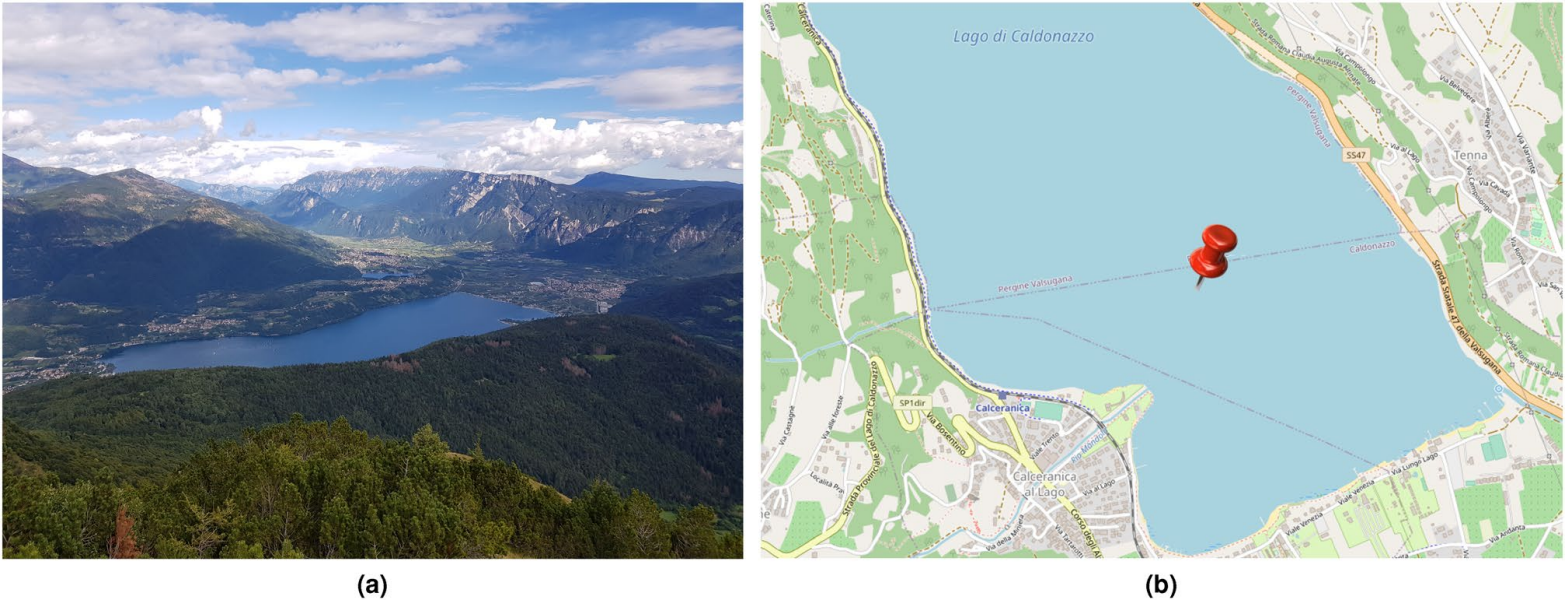
Location of the lake experiment. (**a**) Aerial view of the Caldonazzo lake from the north-west; (**b**) deployment location at the lake’s deepest point (around 43 m). Map courtesy of OpenStreetMap.

### Lake experiment with signals shifted into the transducer’s band

To demonstrate the applicability of our emulation results in a real environment, we conducted a lake experiment, where we transmitted and received the five examined signals using an analog acoustic front-end. The experiment took place in northern Italy, in the Caldonazzo lake (Fig. 6a), on October 18, 2021, in mostly sunny weather with negligible wind. Me moved a 5-m rubber boat to the deepest point of the lake, at coordinates (46.0108386°N, 11.25174835°E, see Fig. 6b). Here, the lake depth is around 43 m. To keep the environment-induced multipath and propagation loss as small as possible, we lowered two EvoLogics mini S2CR modem devices^20^ to a depth of 20 m, one from the front side and another from the aft side of the boat. These devices work in the 18–34 kHz band, and enable the transmission of custom waveforms through a ceramic transducer whose sensitivity and directivity pattern can be found on the manufacturer’s web site^20^. We remark that the receiver side and transmitter side equipment are the same. The modems can record acoustic samples at a rate of 250 ksamples/s.

During the experiment, we repeatedly transmitted biomimicking signals from the front device to the aft device and vice versa. To minimize the impact of multipath reflections originating from structural components of each device, we tuned the transmit power accordingly. To enable the reproducibility of our results, we share the lake experiment dataset with the community^31^. The shared archive contains original signals as well as multiple pre-cut recordings of all vocalizations. We remark that the original signals are the same used also for the emulation-based evaluation above.

**Figure 7:**
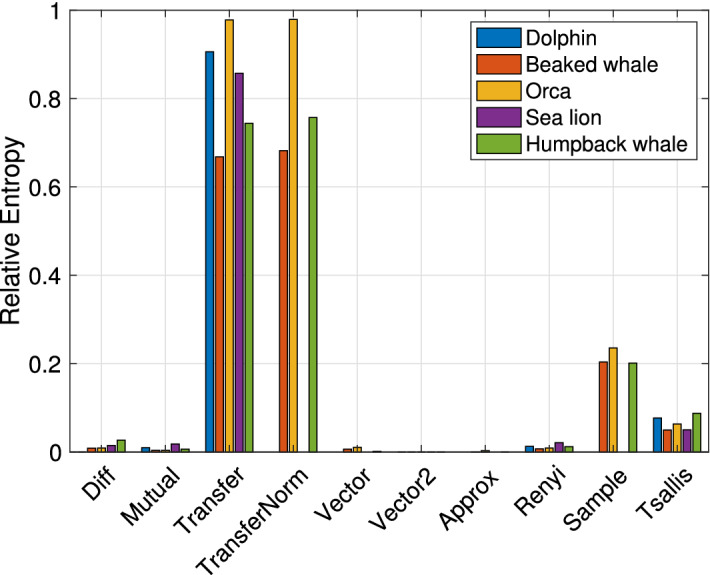
Relative entropy from the lake experiment computed from different entropy metrics, averaged over the 35 transmissions. For all signals, the relative transfer entropy yields a very high discrimination capability.

The results in Fig. 7 show how different entropy measures make it possible to separate the original and the transducer-emitted signal through the computation of their relative entropy. Similar to the emulation results, we observe that the transfer entropy yields the best separation. The results obtained from for the other entropy measures also lead to similar conclusions as the emulation results: for example, the Tsallis entropy, sample entropy, and Renyi entropy also lead to some separation between the original and biomimicking signals, but such separation is much less pronounced than for the transfer entropy. We conclude that our model well reflects the transfer function of a realistic transducer.

Finally, we compare the results in Fig. 7 to examine which marine animal sound leads to the lowest relative entropy, and is thus more suited to for biomimicking acoustic communications. The results show that, similar to the emulation results, the transducer-emitted orca sound is easier to tell apart from its natural version, and cannot be considered a good biomimicking signal. However, we note that the dolphin sound also leads to high relative entropy, whereas the lowest relative entropy values are obtained for the beaked whale sound. These results are in contrast with those of the emulation, because the lake experiment involves a realistic end-to-end transmitter, channel, and receiver chain. Conversely, the emulated results assume ideal behavior for all transceiver electronics except the transducer. Because natural signals have multiple harmonics (see Fig. 3), the relative entropy is influenced not just by the properties of the transducer, but also by the properties of the data acquisition and the power amplifier modules of the receiver.

**Figure 8:**
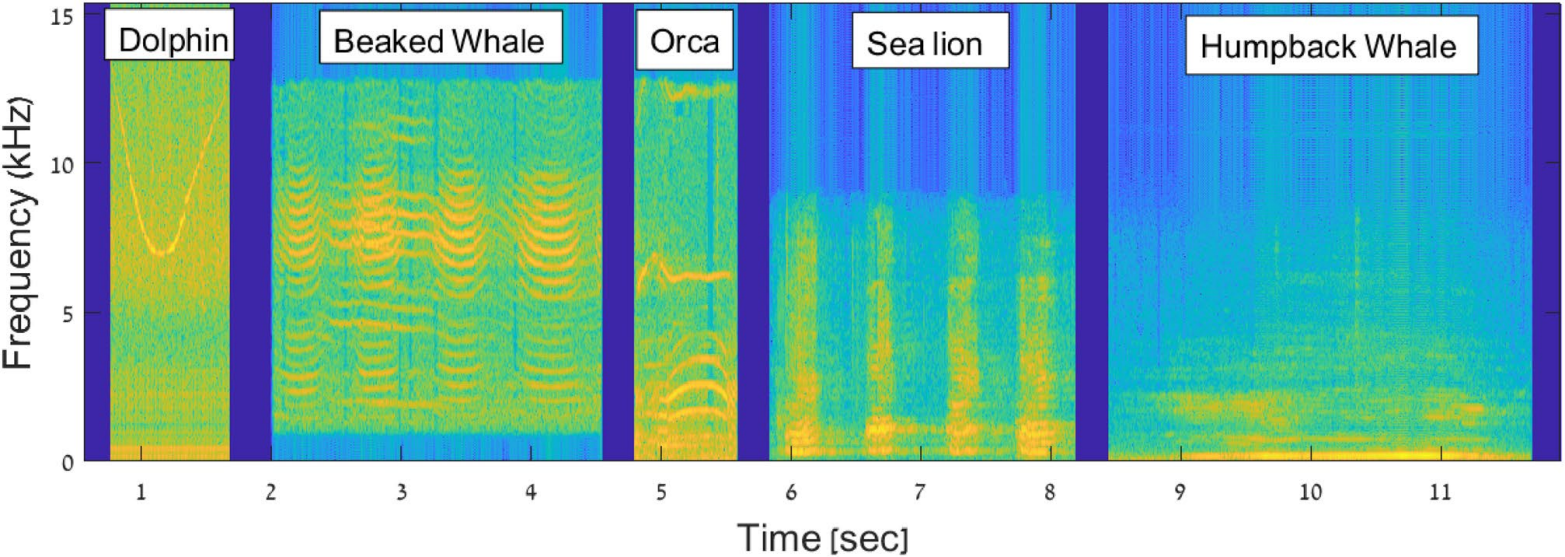
Spectrograms of the (unshifted) bioacoustic signals transmitted during the lake experiment.

### Sea experiment with unshifted vocalizations

In this section, we report the outcomes of the analysis for a sea environment where, different from the lake experiment, ambient noise may affect the entropy results. The sea experiment was performed in Nov. 2022, about 11 km west of Northern Israel, at a water depth of 125 m. An EvoLogics low-frequency ceramic transducer was deployed from a boat at 20 m depth along with a recorder. The receiver’s and transmitter’s hardware are the same, and their sensitivity and directivity pattern can be found on the manufacturer’s web site^32^. A plastic pole was used to align the transducer and recorder, thereby ensuring that the two devices were 1.5 m apart when submerged. Since the transducer can emit signals in the band of 2 kHz-20 kHz, we did not apply any frequency shift to the transmitted signals. The spectra of the emitted, unshifted signals are shown in Fig. 8. As for the lake experiment, emission was made at low power to avoid multipath reflections.

**Figure 9:**
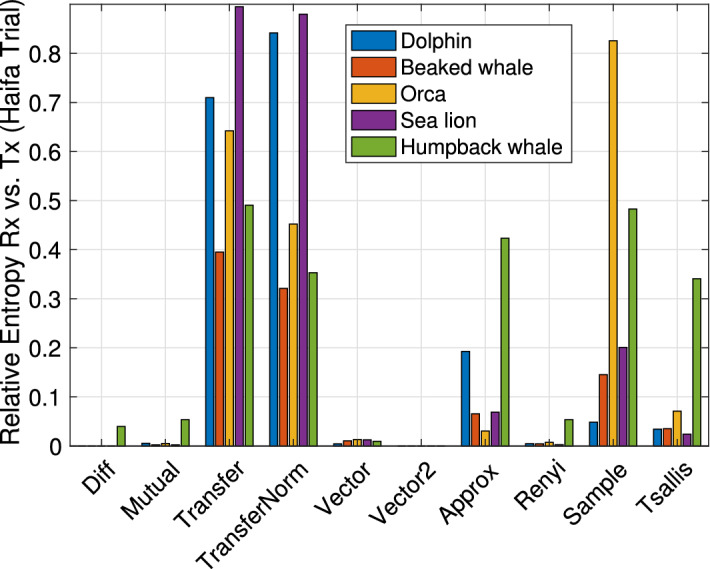
Relative entropy from the sea experiment computed from different entropy metrics, averaged over the 19 transmissions. For all signals, the Transfer entropy yields a very high discrimination capability.

We performed 19 playback repetitions, and computed the average relative entropy for the different entropy metrics. The results are shown in Fig. 9. As in the lake experiment, the transfer entropy metric produces the best separation between the the playback and original signal. We also observe that, as for the lake experiment, the best separation is received for the Orca vocalization. However, the order of separation for the other signals is different than the lake trial. For example, in the sea experiment the second best signal is for the Beaked whale whereas that for the lake trial is the Dolphin whistle. Since in both cases we emitted very low intensity signals and received only the direct propagation path, this result supports our claim that the characteristics of the projector affects the potential of a signal to serve for biomimicking communication.

## Discussion

In this work, we considered biomimicry in underwater acoustic communications, and examined how different marine animal vocalizations lend themselves to be used as biomimicking signals. Our results specifically account for the trans-characteristics of underwater acoustic transducers for underwater digital communications. We show that transducers (whose bandwidth is inherently not as broad as that of a biological vocalization apparatus) tend to have a smoothing effect on transmitted signals. This footprint makes such signals stand apart significantly from animal-emitted sounds, as the latter tend to include wider and more frequent discontinuities. Specifically, we argued that biomimicry can be detected by comparing original animal signals against played-back signals emitted by a transducer, via a relative entropy measure. We also concluded that the entropy metric achieving the best separation between the original and man-made signal is the transfer entropy, which is related to the predictability of a time series.

Our two field experiments emphasize the differences between the properties of a real dolphin’s whistle and its playback from a transducer. Through tests involving two transducers working at different frequency ranges and in different waters (both in a freshwater lake and at sea) we demonstrate that the same conclusion holds for different pieces of hardware. We argue that our results can benefit the research community in two aspects. First, it presents a metric how to intercept biomimicking underwater communications. Different from low-probability-of-detection methods that hide the signal below the ambient noise, biomimicking communications appear plainly to an interceptor, but are typically mistaken as animal vocalizations. In this context, our results can be used to design an interception method, or to explore the performance of a biomimicking communication approach. The second benefit is a new metric for the design of an acoustic transducer. Taking the mammals’ means to vocalize as the ideal case, our entropy metric can be used as another measure for the properties of the transducer. By converting our metric into a *biomimicking*, or *camouflage* rating, the manufacturer of a transducer can evaluate to what extend would the transducer risk to jeopardize biomimicry. To the best of the authors’ knowledge, this is the first work that examines the above effects. Future work includes the explicit modeling of the full electronic front-end chain, including the power amplification and data acquisition stages.

## Data Availability

The experiment dataset analyzed for the current study is available in the at the link https://drive.google.com/file/d/1fH6UNG-7aG90gu7h-8wEjvAVKQyHxxfD/view^31^.
